# Cyclic Nucleotide Dependent Dephosphorylation of Regulator of G-Protein Signaling 18 in Human Platelets

**DOI:** 10.1371/journal.pone.0080251

**Published:** 2013-11-07

**Authors:** Kristina Gegenbauer, Zoltan Nagy, Albert Smolenski

**Affiliations:** 1 UCD Conway Institute, University College Dublin, Dublin, Ireland; 2 UCD School of Medicine and Medical Science, University College Dublin, Dublin, Ireland; 3 National Children’s Research Centre, Crumlin, Dublin, Ireland; 4 Institute of Molecular Medicine, Trinity College Dublin, St James’ Hospital, Dublin, Ireland; University of Leuven, Belgium

## Abstract

Regulator of G-protein signaling 18 (RGS18) is a GTPase-activating protein that turns off Gq signaling in platelets. RGS18 is regulated by binding to the adaptor protein 14-3-3 via phosphorylated serine residues S49 and S218 on RGS18. In this study we confirm that thrombin, thromboxane A2, or ADP stimulate the interaction of RGS18 and 14-3-3 by increasing the phosphorylation of S49. Cyclic AMP- and cyclic GMP-dependent kinases (PKA, PKG) inhibit the interaction of RGS18 and 14-3-3 by phosphorylating S216. To understand the effect of S216 phosphorylation we studied the phosphorylation kinetics of S49, S216, and S218 using Phos-tag gels and phosphorylation site-specific antibodies in transfected cells and in platelets. Cyclic nucleotide-induced detachment of 14-3-3 from RGS18 coincides initially with double phosphorylation of S216 and S218. This is followed by dephosphorylation of S49 and S218. Dephosphorylation of S49 and S218 might be mediated by protein phosphatase 1 (PP1) which is linked to RGS18 by the regulatory subunit PPP1R9B (spinophilin). We conclude that PKA and PKG induced S216 phosphorylation triggers the dephosphorylation of the 14-3-3 binding sites of RGS18 in platelets.

## Introduction

Platelets play important roles in hemostasis, thrombosis and inflammation. Endothelium derived prostacyclin and nitric oxide inhibit platelets by elevating cyclic nucleotide levels in platelets leading to strongly reduced platelet activation, secretion and aggregation. The inhibitory function of cAMP and cGMP is mainly mediated by cyclic nucleotide dependent kinases PKA and PKGI [1], [2]. While a number of PKA and PKGI substrates including VASP, IRAG, Rap1GAP2 and CD-GEFI have been identified in platelets [3], [4], [5], [6], [7], [8], the mechanisms how these proteins translate PKA and PKGI induced phosphorylation into platelet inhibition are not well understood. We recently reported that regulator of G-protein signaling 18 (RGS18) is a new PKA and PKGI substrate in platelets [9]. RGS proteins terminate GPCR signaling by acting as GTPase activating proteins for alpha subunits of heterotrimeric G-proteins [10]. To date, 37 different RGS proteins have been found in humans with at least 11 of these being expressed in platelets [11], [12], [13]. RGS18 is a small RGS of 235 aminoacids belonging to the B/R4 subfamily [11]. RGS18 is highly expressed in platelets and appears to be blood lineage specific and essential for thrombocyte maturation in zebrafish [12], [14], [15], [16], [17], [18]. RGS18 specifically interacts with 14-3-3 protein through phosphorylated S49 and S218 residues [9]. 14-3-3s are phospho-serine/threonine binding adaptor proteins involved in the regulation of signaling pathways [19]. Binding of RGS18 to 14-3-3 is abolished by PKA and PKGI mediated RGS18 phosphorylation on S216 [9]. Loss of 14-3-3 enhances RGS18 function leading to termination of Gq signaling and reduced Ca^2+^ release thus contributing to platelet inhibition. The mechanism underlying PKA/PKGI triggered dissociation of 14-3-3 and RGS18 remain unclear. One possibility is the dephosphorylation of the 14-3-3 binding sites on RGS18 by a phosphatase. 30 catalytic subunits of phosphoserine/threonine phosphatases are known [20]. Targeting and specificity of these phosphatases rely on the formation of holoenzymes with regulatory subunits [21], [22]. For example, the catalytic subunit of protein phosphatase 1 (PP1) is ubiquitously expressed and interacts with more than 90 regulatory subunits [23]. Little is known about the regulation and function of protein phosphatases in platelets.

We show here that cyclic nucleotide mediated phosphorylation of RGS18 results in dephosphorylation of its 14-3-3 binding sites in both resting and activated human platelets. In addition, we provide evidence that this dephosphorylation of RGS18 might be mediated by protein phosphatase 1 and its regulatory subunit PPP1R9B (also known as spinophilin or neurabin-2).

## Materials and Methods

### Ethics Statement

The research involved sampling of venous blood from healthy volunteers who gave their written informed consent in accordance with the principles expressed in the Declaration of Helsinki. This study was approved by the Human Research Ethics Committee of University College Dublin, study number LS-08-13-Smolenski.

### Antibodies

Antibodies against phosphorylated S49 and double phosphorylated S216/S218 of RGS18 were produced using phosphorylated peptides conjugated to keyhole limpet hemocyanin. Immunization of rabbits and subsequent antibody purification were performed by ImmunoGlobe Antikörpertechnik (Himmelstadt, Germany). Antisera were first pre-absorbed on the dephospho peptides, and in the case of the pS216/pS218 antibody also pre-absorbed on singly phosphorylated peptides, and finally affinity purified using the single (pS49) or double (pS216/pS218) phosphorylated target peptides. The antibodies against full-length and phosphorylated S216 of RGS18 have been described [9]. The following commercially available antibodies were used in this study: mouse anti-RGS18 (1H6, Sigma-Aldrich), rabbit anti-RGS18 (Source BioSciences, LS-C785), mouse anti-protein phosphatase 1, catalytic subunit (K19, Santa Cruz). Horseradish peroxidase-coupled donkey anti-rabbit and donkey anti-mouse were from Jackson ImmunoResearch Europe and were used as secondary antibodies for immunoblot analysis visualized by enhanced chemiluminescence (Thermo Scientific). Blots were quantified by densitometry using integrated intensity values (ImageJ program). For detection with Odyssey Infrared Imaging system Western blots were incubated with secondary antibodies IRDye 680LT goat anti-mouse antibody (Li-Cor Biosciences, Cambridge, UK) and IRDye 800CW goat anti-rabbit antibody (Li-Cor, Cambridge, UK). Obtained data was analysed using the Odyssey Application software to obtain values for antibody binding. All statistical analyses were performed using Graphpad Prism 5 software (GraphPad Software, Inc.).

### Constructs

FLAG-tagged RGS18 in mammalian expression vector pcDNA4/TO (Invitrogen) and GST-tagged 14-3-3γ in the bacterial expression vector pGEX-4T3 have been described [9]. An expression vector for myc-tagged spinophilin was provided by Dr. Angus Nairn (Yale University [24]). The catalytic subunit PPP1CA of protein phosphatase 1 was obtained from Source BioScience (clone IRAUp969F0224D) and cloned into pcDNA4/TO with a triple VSV tag. Purified lambda phosphatase and catalytic subunit of PP1 were from New England Biolabs.

### Protein Purification, gel electrophoresis and Phos-Tag gels

GST-14-3-3γ was expressed in *E. coli* BL21 and affinity purified using glutathione-sepharose 4B beads (GE Healthcare). Purity of all proteins was examined by SDS-PAGE followed by Coomassie-Blue staining. SDS-PAGE was conducted using self-cast gels containing 10% acrylamide followed by blotting on nitrocellulose or PVDF (for Phos-tag blots and blots for incubation with phospho-specific antibodies) using semi-dry transfer. For Phos-tag (Wako Chemicals GmbH, Steinbach, Germany) supplemented SDS-PAGE, gels were prepared as described in the manufacturer’s protocol. Briefly, 50 µM Phos-tag and 100 µM MnCl_2_ (Sigma-Aldrich) were added to the separating gel solution prior to polymerization. To remove Mn^2+^ ions before western blotting, gels were incubated in transfer buffer containing 2 mM EDTA (Lennox, Dublin, Ireland) for 15 min at room temperature, followed by an additional wash step in transfer buffer without EDTA for 15 min at room temperature.

### Cell Preparation, Transfection, Lysis, Immunoprecipitation and Pull-down experiments

HEK293T cells were cultured using DMEM supplemented with 10% FCS and 1% penicillin/streptomycin, at 37°C and 5% CO_2_. Cells were transfected using Metafectene (Biontex, Martinsried, Germany) according to manufacturer’s instructions. Platelet isolation was performed as described [25]. Platelets were stimulated at 37°C using either 0.1 U/ml thrombin (Roche) for 30 sec, U-46619 (Cayman Europe) as thromboxane A2 mimetic at 1 µM for 1 min, ADP (Sigma- Aldrich) at 10 µM for 1 min, forskolin at 10 µM for indicated times, prostacyclin (Cayman Europe) at 1 µM for indicated times, sodium nitroprusside (SNP) at 10 µM for 10 min and okadaic acid (Millipore) at 1 µM for 30min. Cell lysis, immunoprecipitation and pull-down assays were performed as indicated using 10 µl of anti-RGS18 monoclonal antibody (Sigma-Aldrich) or 20 µl of rabbit anti-RGS18 antibody followed by 5 µl A/G Sepharose (Santa Cruz), or 1 µl of Gluthatione SepharoseTM 4B suspension (GE Healthcare) saturated with GST-14-3-3γ.

## Results

### Characterization of a phosphorylation site specific antibody against pS49 RGS18

We have recently shown that RGS18 is attached to 14-3-3γ in human platelets. 14-3-3 proteins are dimeric phospho-serine/threonine binding modules that recognize the consensus motifs mode I RSXpS/TXP, mode II RXY/FXpS/TXP or mode III pS/T-X-(X)-COOH [26], [27]. 14-3-3s typically recognize two phosphorylation sites on their target proteins and we identified the binding sites on RGS18 as phosphorylated S218, the dominant site which constitutes a typical mode I motif (RRRSRSFT, with S218 underlined), and phosphorylated S49 on RGS18, a site of lesser affinity which does not fully correspond to any of the classic 14-3-3 binding motifs (KRNRLSLL, with S49 underlined) [9]. We could also show that PKA and PKGI mediated phosphorylation of S216 on RGS18 inhibits binding to 14-3-3 in human platelets [9]. These results lead to the question how S216 phosphorylation might induce detachment of 14-3-3 from RGS18. We envisioned three possible mechanisms: (i) pS216 interferes directly with the interaction of RGS18 and 14-3-3, (ii) pS216 induces the dephosphorylation of pS218 and/or pS49, or (iii) the kinase responsible for phosphorylating the 14-3-3 binding sites is inhibited by cyclic nucleotide elevation.

**Figure 1:**
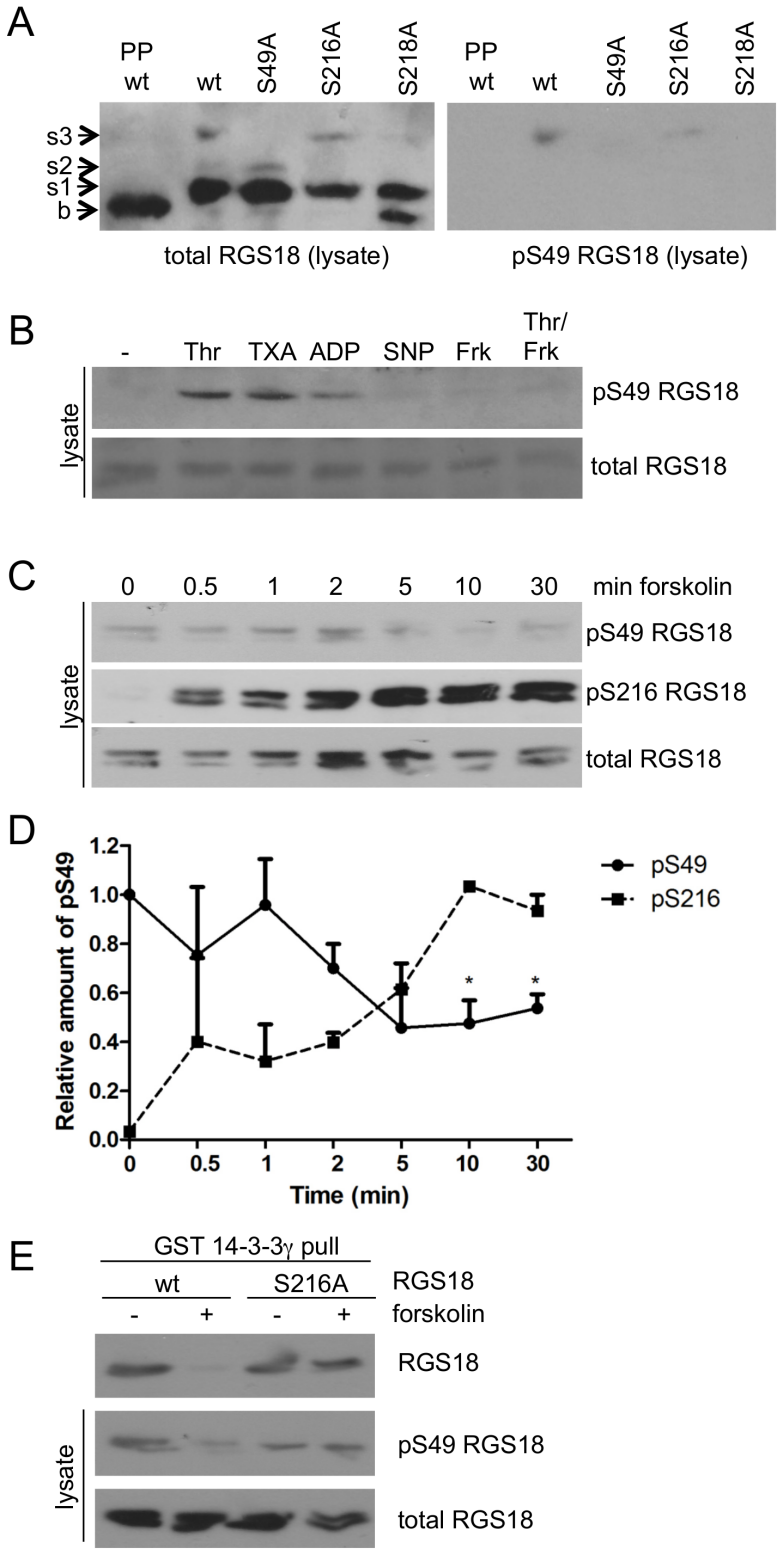
Dephosphorylation of pS49 of RGS18. A. Characterization of an anti-pS49 RGS18 antibody. FLAG-tagged wt and point mutants (as indicated) of RGS18 were expressed in HEK293T cells. Cells were lysed, one sample of wt-expressing cells was incubated with lambda phosphatase for 1h, and all samples were subjected to Phos-tag supplemented SDS-PAGE and Western blotting using mouse anti-total RGS18 antibody (left panel) followed by rabbit anti-pS49 RGS18 antibody (right panel). Appearing bands were labelled as baseline (‘b’), shift 1 (‘s1’), shift 2 (‘s2’), and shift 3 (‘s3’) with increasing apparent molecular weight. B. S49-RGS18 phosphorylation in intact human platelets. Washed platelets were incubated without or with 0.1 U/ml thrombin for 30sec, 1 µM U46619 or 10 µM ADP for 1 min, 10 µM sodium nitroprusside for 10min, 10 µM forskolin for 30min, or a combination of forskolin and thrombin. After lysis and SDS-PAGE, blots were incubated with either anti-pS49 RGS18 (upper panel) or rabbit anti-RGS18 (lower panel). C. Time-course of S49 and S216 phosphorylation of RGS18 in HEK293T cells. Wt-RGS18 transfected HEK293T cells were stimulated with 10 µM forskolin for the indicated times. Cells were then lysed and lysates were subjected to SDS-PAGE and Western blotting using anti-pS49 RGS18 (top panel), rabbit anti-pS7 Rap1GAP2 which detects S216 RGS18 (middle panel) and mouse anti-RGS18 (lower panel). Shown is a representative example of experiments performed three times. D. Quantitation of pS49 and pS216 signals shown in C. Band intensities were determined by densitometry and ratios of pS49-RGS18 or pS216-RGS18 to total RGS18 were calculated. Values were normalized to 0 min forskolin stimulation (pS49) or highest signal (pS216) and presented as means +SEM. Statistical significance in relation to the ‘0’ timepoint was determined using paired Students t-test, *p<0.05. E. Role of S216 for dephosphorylation of pS49 and loss of RGS18 binding to 14-3-3. FLAG-tagged wt- and S216A mutant RGS18 were expressed in HEK293T cells. Cells lysates were subjected to GST-14-3-3γ pull-down. Western blots of precipitates were incubated with mouse anti-RGS18 (upper panel) and lysate blots with rabbit anti-pS49 RGS18 (middle panel) or mouse anti-RGS18 as loading control (lower panel).

To address these mechanisms we generated an antibody against phosphorylated S49 of RGS18 and used RGS18 mutants and Phos-tag SDS-PAGE to validate its specificity. Western blotting of wt RGS18 samples expressed in HEK293T cells using Phos-tag gels and a common non-phospho antibody against RGS18 revealed four bands which we termed baseline, shift 1, shift 2 and shift 3, as before (Figure 1A, left panel) [9]. The s3 band appeared only in wt and S216A mutant RGS18 constructs in HEK293T cells while in S49A mutant RGS18 the s3 band was lacking (Figure 1A, left panel, and [9]). Incubation with the pS49 RGS18 antibody showed only bands corresponding to s3 in wt and S216A RGS18 confirming the specificity of the antibody as well as the identity of s3 as pS49 (Figure 1B, right panel). Interestingly, the pS49 band disappeared also in the S218 mutant suggesting that S49 phosphorylation might depend on S218. The s3/pS49 band was sensitive to treatment of samples with the serine/threonine/tyrosine phosphatase lambda further confirming its identity as a phosphorylation site. Treatment of platelets with thrombin, thromboxane, or ADP induced the phosphorylation of S49 of RGS18 (Figure 1B, lanes 2-4). Phosphorylated S49 has also been detected in response to TRAP in a proteome analysis of activated platelets [28]. Activation of cyclic nucleotide-dependent signaling with sodium nitroprusside, a NO-donor, or forskolin, an activator of adenylyl cyclase, did not induce S49 phosphorylation (Figure 1B, lanes 5, 6). Furthermore, forskolin treatment abolished thrombin induced S49 phosphorylation (Figure 1B, last lane).

### Phosphorylation of S216 induces dephosphorylation of S49 of RGS18

We sought to examine S49 phosphorylation dynamics more closely in a forskolin time-course of wt RGS18 transfected HEK293T cells. To control for successful forskolin stimulation, lysates were incubated with anti-pS216 RGS18 antibody (Figure 1C, middle panel). After about 2 to 5 min of forskolin treatment pS49 phosphorylation started to decrease (Figure 1C, top panel and Figure 1D) reaching significant levels after 10 min (Figure 1D). This result was consistent with data from activated platelets (Figure 1B). This data indicated that pS49 on RGS18 was either dephosphorylated in response to cAMP elevation and S216 phosphorylation or that the kinase responsible for phosphorylating S49 was inhibited by cyclic nucleotide elevation. To address these alternatives, we expressed wild-type and S216A mutant RGS18, which cannot be phosphorylated by PKA [9], and performed GST-14-3-3 pull-down experiments after forskolin treatment. cAMP elevation did not affect S49 phosphorylation and binding of the S216A mutant RGS18 to 14-3-3 (Figure 1E). These data suggest that dephosphorylation of pS49 and removal of 14-3-3 depend on the presence of phosphorylatable S216, whereas involvement of another kinase appears less likely.

### Phosphorylation of S216 induces dephosphorylation of S218 of RGS18 in transfected cells

**Figure 2:**
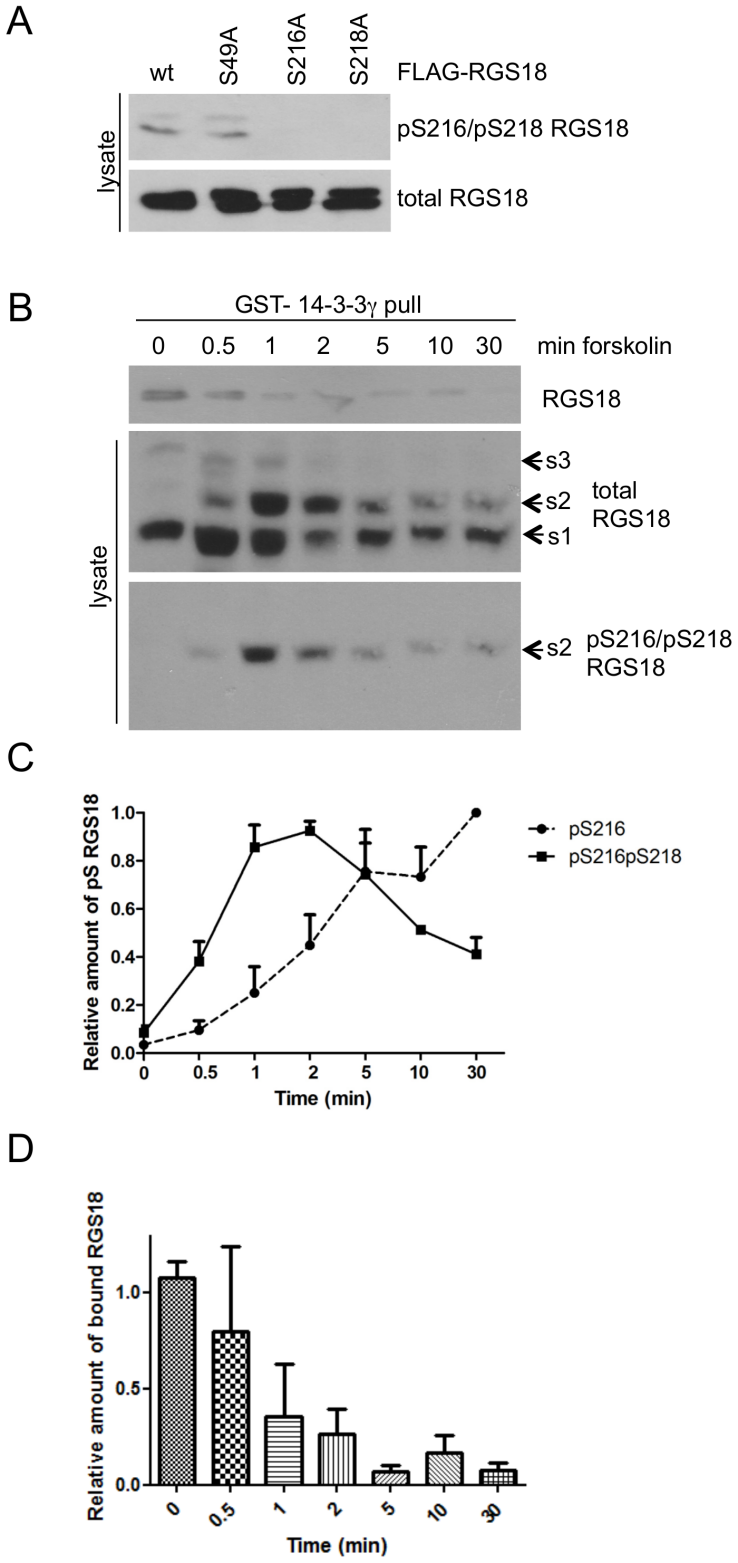
Dephosphorylation of pS218 of RGS18 in HEK293T cells. A. Characterization of a pS216pS218 RGS18 double-phospho antibody. FLAG-tagged wt-and point mutants of RGS18 were expressed in HEK293T cells and analyzed by Western blotting using rabbit anti-pS216pS218 RGS18 antibody (upper panel) and subsequently with mouse anti-RGS18 as loading control (lower panel). B. Time-course of S216/S218 phosphorylation and binding to 14-3-3. Wt-RGS18 was expressed in HEK293T cells and before lysis cells were treated with 10 µM forskolin for indicated times. GST-14-3-3γ pull-down assays were performed and precipitates were analyzed by Western blotting using mouse anti-RGS18 (upper panel). Lysates were subjected to Phos-tag supplemented SDS-PAGE and Western blotting using rabbit anti-pS218pS216 RGS18 (lower panel) and subsequently with mouse anti-RGS18 (middle panel). C. Evaluation of three independent experiments for pS216/pS218 (B) and pS216 (blots not shown). Blots were quantified by densitometry and relative signals of pS216/pS218 and pS216 RGS18 were normalized to the highest value. To control for loading, these values were divided by the sum of s1, s2 and s3 bands of the corresponding total RGS18 lanes, normalized to the 0 min time point. Shown are means +SEM. D. Quantitation of results shown in B, top panel. Blots of 2 independent experiments were analyzed by densitometry and relative intensities of precipitated 14-3-3 bound RGS18 versus total RGS18 are presented as means +SEM.

To examine the phosphorylation state of S218 in response to forskolin treatment, we developed an antibody that recognized the double phosphorylated pS216/pS218 site. To validate the specificity of the antibody we used RGS18 mutants and Phos-tag gels. Mutation of either S216 or S218 abolished binding of the pS216/pS218 antibody to RGS18 (Figure 2A). In Phos-tag gels of lysates from wt-RGS18 transfected HEK293T cells treated with forskolin the pS216/pS218 antibody selectively recognized and followed the time-dependent pattern of the s2 shift (Figure 2B, lower panel). These findings confirm the previously made suggestion that the s2 shift represents double phosphorylated RGS18 [9]. Comparison of the kinetics of single S216 and double S216/S218 phosphorylation showed that single pS216 increased continuously throughout the time course (Figure 2C, and 1C, D). In contrast, the pS216/pS218 signal reached a peak at about 1–2 min followed by a continuous decline (Figure 2B and C) indicating a loss of S218 phosphorylation. This loss of pS218 was matched by a reduction of RGS18 binding to 14-3-3 (Figure 2B, top panel, quantified in 2D). Interestingly, the reduced RGS18 binding was evident already during the peak of pS216/pS218 double phosphorylation after 1-2 min of forskolin treatment. A number of conclusions can be drawn from these experiments: (i) pS218 is present in the basal state and contributes to binding of RGS18 to 14-3-3, (ii) PKA induced phosphorylation of S216 in the presence of pS218 impairs binding of RGS18, and (iii) continued S216 phosphorylation triggers the dephosphorylation of pS218 resulting in reduced binding of RGS18.

### Phosphorylation of S216 induces dephosphorylation of S218 of RGS18 in human platelets

**Figure 3:**
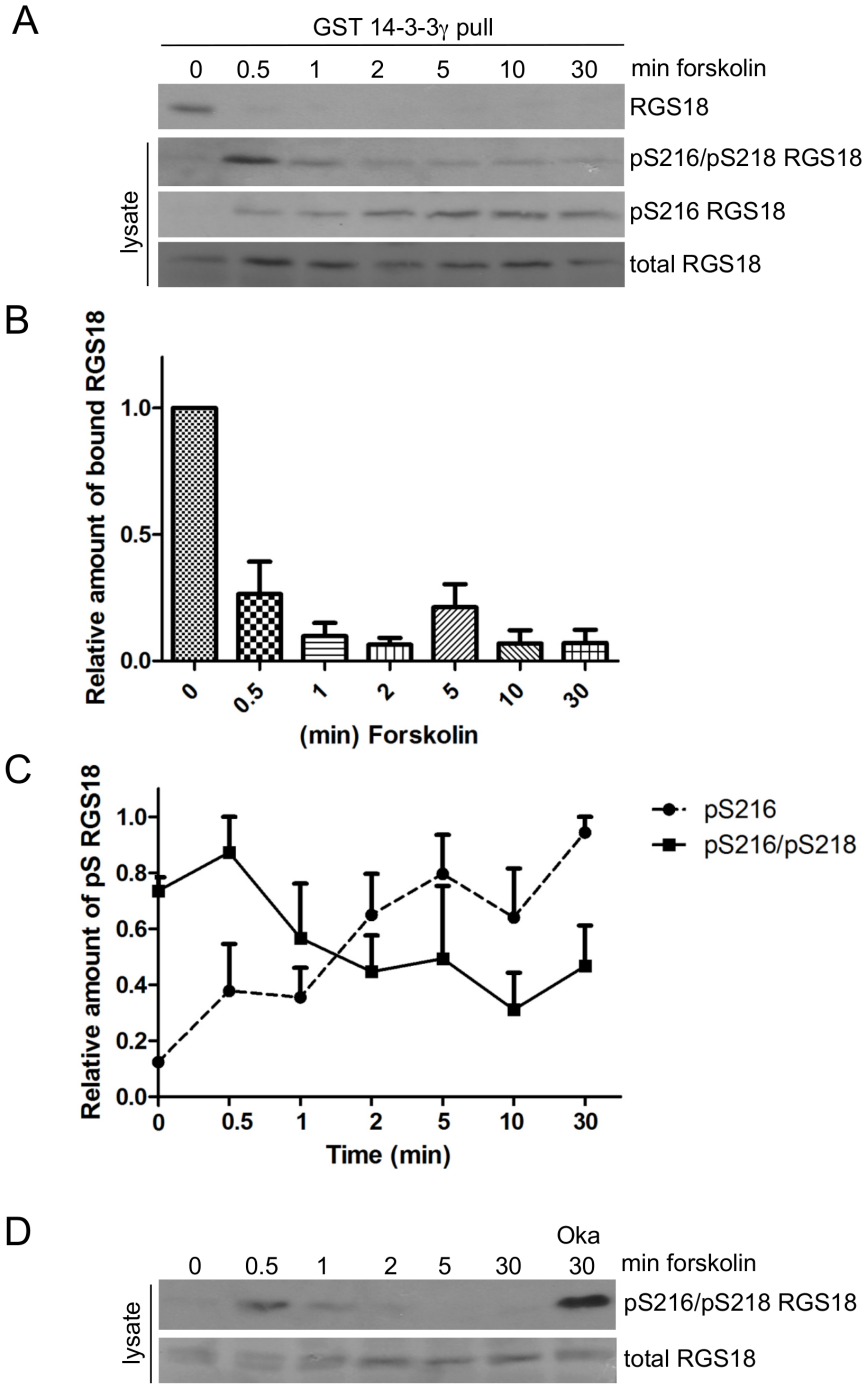
Dephosphorylation of pS218 of RGS18 in human platelets treated with forskolin. A. Time-course of S216 and S218 phosphorylation and binding to 14-3-3. Washed platelets were incubated without or with 10 µM forskolin for the indicated times. Platelets were lysed and lysates were subjected to GST-14-3-3γ pull-down. Precipitates were analyzed by Western blotting using mouse anti-RGS18 antibody (upper panel, RGS18). Lysate blots were incubated with either rabbit anti-pS216pS218 RGS18 (second panel), rabbit anti pS216 RGS18 (third panel) or rabbit anti-RGS18 (lower panel, total RGS18). Shown is a representative example of four independent experiments. B. Quantitation of results shown in A, top panel. Blots of 4 independent experiments were analyzed by densitometry and relative intensities of precipitated RGS18 versus total RGS18 are presented as means +SEM. C. Quantitation of results shown in A, pS216pS218 and pS216 RGS18 panels. Band intensities were determined by densitometry and ratios of pS216/pS218 RGS18 or pS216RGS18 to total RGS18 were calculated. Values were normalized to highest values and presented as means +SEM. D. Effect of phosphatase inhibition on S216/S218 phosphorylation. Washed platelets were incubated without or with 10 µM forskolin or 1 µM okadaic acid as indicated. Platelets were lysed and lysates were subjected to SDS-PAGE and Western blotting. Blots were incubated with either rabbit anti-pS216pS218 RGS18 (upper panel) or rabbit anti-RGS18 (lower panel).

To investigate the kinetics of S216 and S218 phosphorylation at endogenous protein levels we studied human platelets. Forskolin treatment resulted in a time-dependent increase in S216 phosphorylation starting after 0.5 min (Figure 3A, third panel and Figure 3C/, pS216) whereas double phosphorylation of S216/S218 declined (Figure 3A, second panel and Figure 3C, pS216/pS218). In addition, binding to 14-3-3 was reduced after 0.5 min of forskolin stimulation (Figure 3B), coinciding with the highest pS216/pS218 signal (Figure 3A, top and second panel and Figure 3C, pS216/pS218). This result confirmed that the phosphorylation of S216 in itself already inhibited binding of pS218 to 14-3-3. Continued phosphorylation of S216 lead to dephosphorylation of pS218 and detachment of RGS18 from 14-3-3 in human platelets. To test the possible role of phosphatases in forskolin-induced dephosphorylation of pS218, we used the non-specific serine/threonine phosphatase inhibitor okadaic acid. Okadaic acid treatment caused a strong increase in the pS216/pS218 RGS18 signal (Figure 3D) indicating that a phosphatase might be involved in the reduction of S218 phosphorylation.

**Figure 4:**
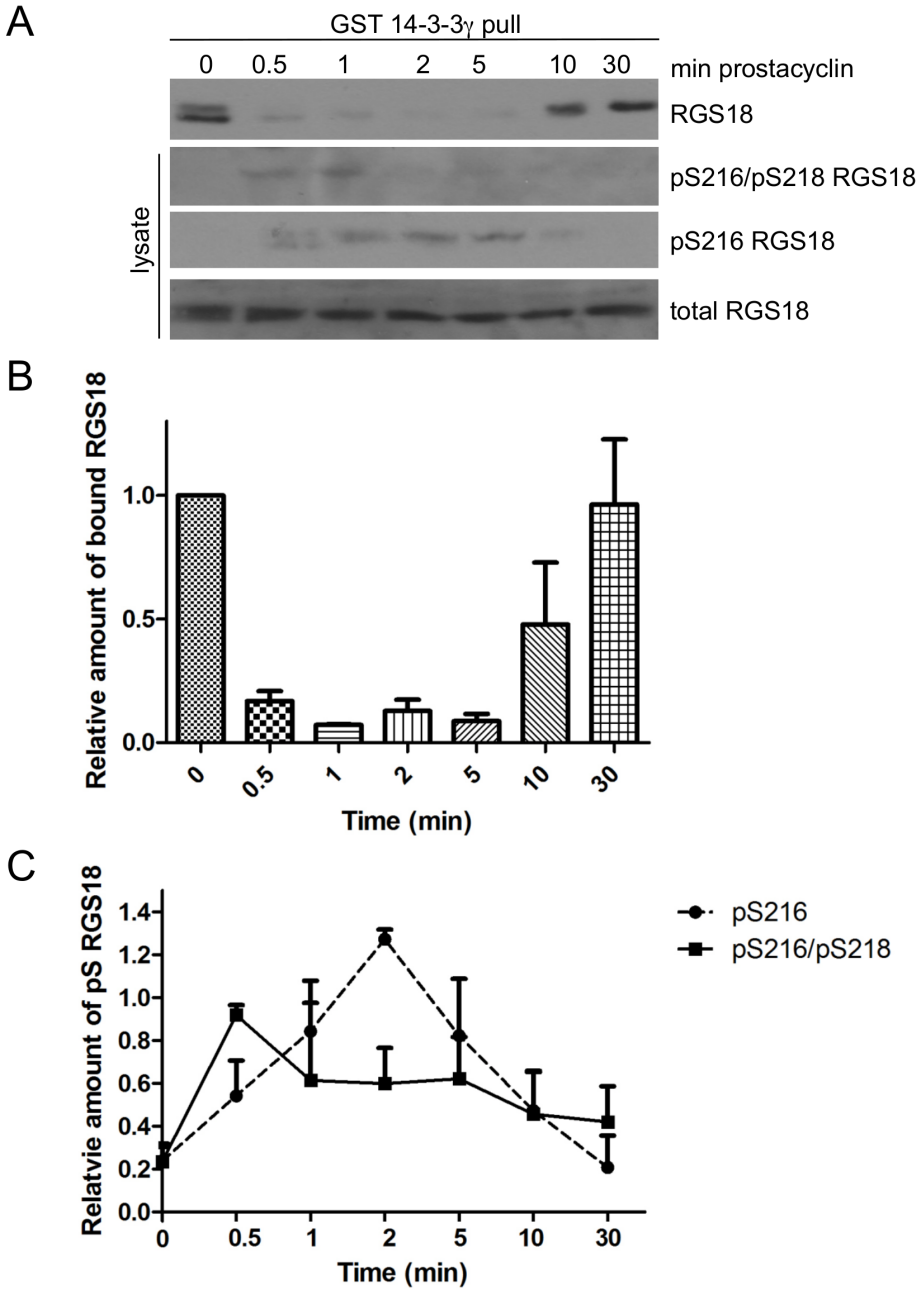
Dephosphorylation of pS218 of RGS18 in human platelets treated with prostacyclin. A. Time-course of S216 and S218 phosphorylation and binding to 14-3-3. Washed platelets were incubated without or with 1 µM prostacyclin as indicated. Platelet lysates were subjected to GST-14-3-3γ pull-down. Precipitates were analyzed by Western blotting using mouse anti-RGS18 (upper panel). Lysate blots were incubated with either rabbit anti-pS216pS218 RGS18 (second panel), rabbit anti pS216 RGS18 (third panel) or rabbit anti-RGS18 (lower panel). Shown is a representative example of three independent experiments. B. Quantitation of results shown in A, top panel. Blots of 3 independent experiments were analyzed by densitometry and relative intensities of precipitated RGS18 versus total RGS18 are presented as means +SEM. C. Quantitation of results shown in A, pS216pS218 and pS216 RGS18 panels. Band intensities were determined by densitometry and ratios of pS216/pS218 RGS18 or pS216RGS18 to total RGS18 were calculated. Values were normalized to highest values and presented as means +SEM.

We next tested whether receptor-mediated elevation of cAMP had a similar effect on RGS18 dephosphorylation and binding to 14-3-3 as forskolin, a direct activator of adenylyl cyclase. For this we used prostacyclin, the agonist of the Gs-coupled prostacyclin receptor in human platelets. Prostacyclin treatment induced a transient phosphorylation of S216 lasting from 0.5 min to about 5 min, which can be explained by the short half-life of prostacyclin at neutral pH (Figure 4A, third panel and Figure 4C, pS216). The pS216/pS218 signal reached its maximum at 0.5 min followed by a continuous decline (Figure 4A, second panel and Figure 4C, pS216/pS218). Importantly, binding of RGS18 to 14-3-3 correlated inversely with the pS216 signal. RGS18 binding was strongly reduced after 0.5 min and was restored after 10 min of prostacyclin stimulation coinciding with decreasing levels of pS216 (Figure 4A, upper panel and Figure 4B). We conclude that PKA-mediated S216 phosphorylation interfered with binding of pS218 to 14-3-3 at early time points (0.5 and 1 min). Prolonged S216 phosphorylation induced the dephosphorylation of pS218 precluding the ability of RGS18 to bind 14-3-3. These data also indicated that the process of S218 dephosphorylation and detachment of RGS18 from 14-3-3 is readily reversible. Loss of prostacyclin induced cAMP signaling lead to the dephosphorylation of S216 and restored the RGS18/14-3-3 interaction (Figure 4A, B, 10 and 30 min time points). These data confirm the findings obtained using forskolin in transfected HEK293T cells (Figure 2) and in platelets (Figure 3A-C).

### Involvement of protein phosphatase 1 in the dephosphorylation of RGS18

**Figure 5:**
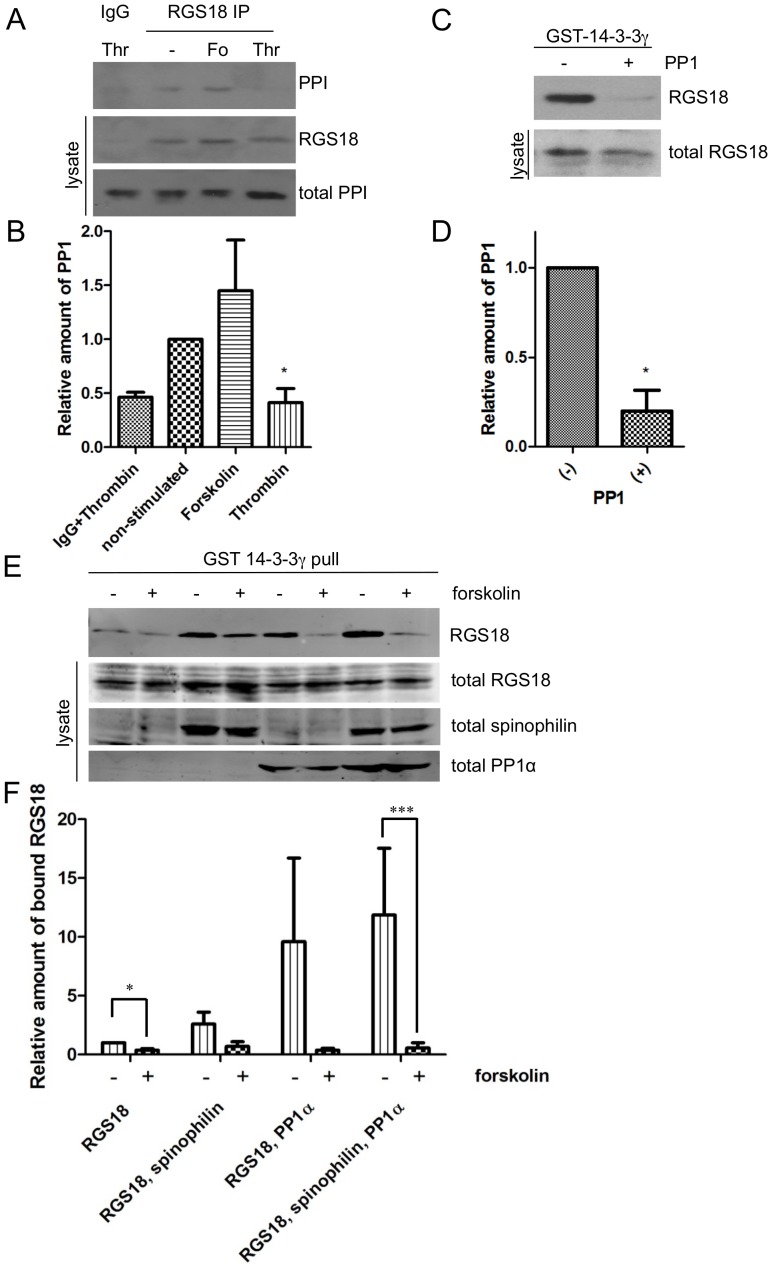
Role of protein phosphatase 1 in the dephosphorylation of RGS18. A. Coprecipitation of catalytic subunit of protein phosphatase 1 (PP1) and RGS18 in platelets. Washed human platelets were incubated without or with forskolin (10 µM, 30min) or thrombin (0.1 U/ml, 30sec). Platelets were lysed and endogenous RGS18 was precipitated with rabbit anti-RGS18 antibody (LS-Bio). After SDS-PAGE and Western blotting, blots of precipitates were incubated with mouse anti-PP1 (upper panel, PP1) and subsequently with mouse anti-RGS18 (middle panel). Lysate blots were incubated with mouse anti-PP1 (lower panel, total PP1). B. Quantitation of results shown in A. Relative intensities of PP1 bands were normalized to the non-stimulated value. To control for loading, these values were divided by corresponding total PP1 and RGS18 values, which had also been normalized to the non-stimulated value. Shown are means +SEM of three independent experiments. Statistical significance was assessed using paired Students t-test comparing non-stimulated and thrombin treated samples, *p<0.05. C. PP1 abrogates binding of RGS18 to 14-3-3. Lysates of non-treated washed platelets were subjected to GST-14-3-3γ pull-downs. Precipitates were incubated for 2h without or with catalytic subunit of PP1 followed by Western blotting using mouse anti-RGS18 (upper panel). Western blots of lysates were incubated with rabbit anti-RGS18 (lower panel). Shown is a representative of 3 independent experiments. Statistical significance was assessed using paired Students t-test comparing non-treated and PP1 treated GST-14-3-3γ pull-downs, *p<0.05. D. Densitometric evaluation of data shown in C. Intensities of RGS18 bands were normalized to the non-treated value and divided by total RGS18 intensities to control for loading. Shown are means +SEM. E. PP1 overexpression enhances the forskolin-induced reduction of RGS18 binding to 14-3-3. VSV-tagged catalytic subunit of PP1 and myc-tagged spinophilin were co-expressed together with FLAG-tagged RGS18 construct in HEK293T cells as indicated. Cells were incubated without or with 10 µM forskolin for 15 min, lysed and pull-down assays were performed using purified GST-14-3-3γ. Binding of RGS18 to 14-3-3 was evaluated by Western blotting using mouse anti-FLAG antibody (top panel, RGS18). Expression levels of transfected constructs were assessed by blotting of total cell lysates using rabbit anti-RGS18 (second panel, total RGS18), mouse anti-myc (third panel, total spinophilin), and mouse anti-VSV (bottom panel, total PP1). IRDye labelled secondary antibodies were used for detection with the Odyssey Imaging system. F. Quantitative evalulation of E. Blots of three independent experiments shown in E were quantified using Odyssey software. To control for loading, 14-3-3 bound RGS18 values were divided by total RGS18 values and normalized to non-treated RGS18 only expressing samples. Differences in 14-3-3 binding between non-treated and forskolin-treated samples were statistically significant (paired Students t-test, *p<0.05, ***p<0.001).

Recently, the adaptor protein spinophilin was shown to interact with RGS18 in human platelets [29]. Spinophilin is also known as regulatory subunit PPP1R9B of PP1, however, PP1 binding to spinophilin and RGS18 has not been studied in platelets so far. To investigate whether PP1 might be linked to RGS18 via spinophilin, we treated human platelets with either forskolin or thrombin and performed RGS18 immunoprecipitations. PP1 coprecipitated with RGS18 in non-stimulated and forskolin treated platelets but dissociated upon thrombin stimulation (Figure 5 A, B). Dissociation of PP1 from the RGS18 complex was expected because spinophilin has been shown before to detach from RGS18 upon thrombin treatment [29]. Thus loss of PP1 might reflect the loss of spinophilin. To test whether PP1 might be involved in the dephosphorylation of RGS18 on pS218, we performed GST-14-3-3γ pull-down experiments using lysates of resting human platelets and subsequently treated the pull-downs containing RGS18 with purified catalytic subunit of PP1. Indeed, PP1 treatment abolished RGS18 binding to 14-3-3 (Figure 5C and D), confirming that PP1 was able to dephosphorylate the 14-3-3 binding sites on RGS18. To address the potential role of PP1 in intact cells we co-expressed RGS18, spinophilin, and the PP1 catalytic subunit in HEK293T cells and performed GST-14-3-3 pull-down assays. Coexpression of RGS18 with spinophilin and/or PP1 increased binding of RGS18 to 14-3-3 (non-treated ‘-‘ lanes in Figure 5E, upper panel) suggesting that spinophilin and PP1 stabilize the interaction of RGS18 and 14-3-3 possibly by formation of a trimeric spinophilin/PP1/RGS18 complex. The ability of PP1 alone to increase RGS18 binding might be due to recruitment of endogenous spinophilin which has been shown to be expressed in HEK293T cells [30] (Figure 5E). Importantly, we observed differences in the capacity of forskolin to reduce RGS18 binding to 14-3-3 depending on the presence of spinophilin and PP1 (treated ‘+’ lanes in Figure 5E, upper panel, and quantitation in 5F). Forskolin treatment reduced RGS18 binding in all samples as seen before (Figure 1E). However, considering the higher baseline levels of RGS18 binding in these cells the relative reduction of RGS18 binding was more pronounced in cells expressing PP1. Using baseline, non-treated samples as reference forskolin was able to reduce RGS18 binding to 14-3-3 by 2.6-fold in cells expressing RGS18 alone (first two bars in Figure 5F), 3.7-fold in cells expressing RGS18 and spinophilin (bars 3, 4), 25-fold in cells expressing RGS18 and PP1 (bars 5, 6), and 21-fold in cells expressing all three proteins which was highly significant (bars 7, 8). These data suggest that the presence of PP1 and spinophilin enhances the detachment of 14-3-3 from RGS18 possibly by facilitating the dephosphorylation of pS218 and pS49. Thus, PP1 might mediate the dephosphorylation of the 14-3-3 binding sites pS49 and pS218 of RGS18 in response to cyclic nucleotide mediated phosphorylation of S216.

## Discussion

**Figure 6:**
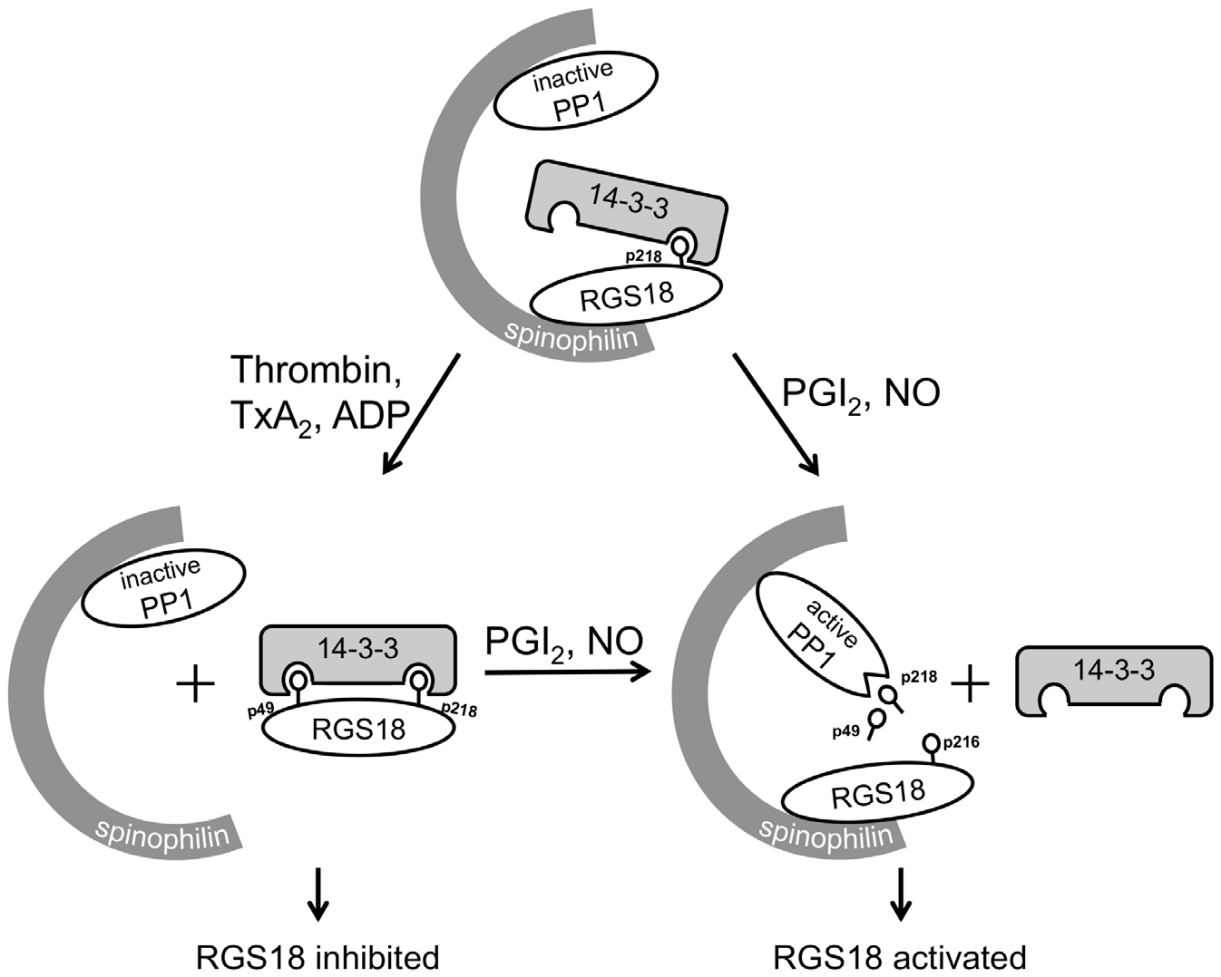
Model of the regulation of the RGS18 complex in platelets. In resting platelets RGS18 is attached to the scaffold protein spinophilin which also binds PP1, and the tyrosine phosphatase SHP-1 (not shown). In addition, RGS18 binds 14-3-3 via phosphorylated S218 of RGS18 (top). Platelet activation by thrombin, thromboxane A2, or ADP leads to the phosphorylation of S49 and increased 14-3-3 binding. Thrombin also induces the detachment of spinophilin together with PP1, which might prevent dephosphorylation of the 14-3-3 binding sites pS49 and pS218. 14-3-3 reduces the function of RGS18 resulting in facilitated Gq signaling which contributes to platelet activation. In contrast, activation of cAMP- and cGMP-dependent protein kinases by prostacyclin (PGI_2_) or nitric oxide (NO) leads to the phosphorylation of RGS18 on serine 216 (pS216). S216 phosphorylation might activate PP1 leading to dephosphorylation of both 14-3-3 binding sites, S49 and S218, and detachment of 14-3-3. Removal of 14-3-3 activates RGS18 to turn off Gq signaling thus contributing to platelet inhibition.

The RGS18/14-3-3 interaction is regulated by a series of distinct phosphorylation and dephosphorylation events. Binding of RGS18 to 14-3-3 requires phosphorylated S218 and can be further enhanced by phosphorylation of S49 during platelet activation by thrombin, thromboxane A2, or ADP (Figure 6). On the other hand cyclic nucleotide inhibitory pathways disrupt the RGS18/14-3-3 interaction by phosphorylating S216. We show that S216 phosphorylation has two effects: (i) pS216 interferes with binding of pS218 to 14-3-3, and (ii) pS216 triggers the dephosphorylation of pS49 and pS218. Our data suggest that phosphorylation of the S216 site, which is at the -2 position of the 14-3-3 binding site S218, impairs binding of phosphorylated S218 to 14-3-3. Interestingly, phospho-mimetic amino acids like aspartic or glutamic acid are almost completely disfavored at the -2 position of 14-3-3 binding sites suggesting an inhibitory effect of negative charges at this position on 14-3-3 binding [31]. The second mechanism mediating detachment of 14-3-3 involves dephosphorylation of the 14-3-3 binding sites pS49 and pS218 of RGS18. Our studies of the kinetics of S49, S216, and S218 phosphorylation in HEK293T cells and human platelets indicate that S216 phosphorylation precedes the dephosphorylation of pS49 and pS218.

We provide evidence that the dephosphorylation of both 14-3-3 binding sites on RGS18 in response to cAMP elevation might involve PP1 and its regulatory subunit spinophilin: purified PP1 is able to remove 14-3-3 from RGS18 (Figure 5C), the PP1 inhibitor okadaic acid prevents the dephosphorylation of RGS18 (Figure 3D), PP1 and spinophilin facilitate the detachment of 14-3-3 in transfected cells (Figure 5E), and PP1 and RGS18 coprecipitate in platelets which is regulated by thrombin (Figure 5A). This model is supported by a recent study showing a complex of RGS18, spinophilin, and the tyrosine phosphatase SHP-1 in platelets although the presence of PP1 in this complex was not investigated [29]. The role of spinophilin as a regulatory subunit of PP1 has been established by numerous studies including structural analysis [32], [33]. Similar mechanisms of regulation of 14-3-3 binding have been shown before for the protein phosphatases Cdc25B and Cdc25C, p53, and for the GTPase-activating protein Rap1GAP2 [34], [35], [36]. For example, phosphorylation of the -2 serine relative to a phosphorylated 14-3-3 binding site removes 14-3-3, leads to recruitment of PP1 and decreased phosphorylation of the 14-3-3 binding site on Cdc25 [37], [38]. The exact mechanism involved in PP1 activation leading to dephosphorylation of the 14-3-3 binding sites pS49 and pS218 on RGS18 after S216 phosphorylation remains to be determined. Crystallographic data on the isolated spinophilin-PP1 complex suggests that spinophilin can direct PP1 catalytic activity by affecting its substrate binding pockets without altering its catalytic site [33]. The resolution of the structural consequences of PKA induced RGS18 phosphorylation and 14-3-3 detachment on the RGS18-spinophilin-PP1 complex may provide insights into PP1 regulation and substrate specificity. Interestingly, cyclic nucleotide effects on binding of RGS18 to 14-3-3 are reversible since platelets can restore the ability of RGS18 to bind 14-3-3 after elevated cAMP levels have dropped (Figures 4, 6). This suggests that as the activity of PKA is reduced, pS216 is dephosphorylated which might possibly also be mediated by PP1. Subsequently, RGS18 is again phosphorylated on S218 by an unknown kinase, resulting in 14-3-3 re-association.

This study indicates that secondary dephosphorylation events might play an important role in mediating the powerful inhibitory actions of cyclic nucleotide signaling pathways in platelets.
